# Pigment and Fatty Acid Heterogeneity in the Sea Slug *Elysia crispata* Is Not Shaped by Habitat Depth

**DOI:** 10.3390/ani11113157

**Published:** 2021-11-05

**Authors:** Xochitl Guadalupe Vital, Felisa Rey, Paulo Cartaxana, Sónia Cruz, Maria Rosário Domingues, Ricardo Calado, Nuno Simões

**Affiliations:** 1 Posgrado en Ciencias Biológicas, Unidad de Posgrado, Edificio D, 1° Piso, Circuito de Posgrados, Ciudad Universitaria, Alcaldía Coyoacán, Ciudad de México 04510, Mexico; vital@ciencias.unam.mx; 2 UMDI-Sisal, Facultad de Ciencias, Universidad Nacional Autónoma de México, Puerto de Abrigo S/N, Sisal 97356, Mexico; 3 Centre for Environmental and Marine Studies (CESAM), Department of Chemistry, Campus Universitário de Santiago, University of Aveiro, 3810-193 Aveiro, Portugal; mrd@ua.pt; 4 Mass Spectrometry Centre & LAQV-REQUIMTE, Department of Chemistry, Campus Universitário de Santiago, University of Aveiro, 3810-193 Aveiro, Portugal; 5 ECOMARE, CESAM, Department of Biology, Campus Universitário de Santiago, University of Aveiro, 3810-193 Aveiro, Portugal; pcartaxana@ua.pt (P.C.); sonia.cruz@ua.pt (S.C.); rjcalado@ua.pt (R.C.); 6 International Chair for Coastal and Marine Studies, Harte Research Institute for Gulf of Mexico Studies, Texas A and M University-Corpus Christi, Corpus Christi, TX 78412, USA; 7 Laboratorio Nacional de Resiliencia Costera (LANRESC), Laboratorios Nacionales, CONACYT, Sisal 97356, Mexico

**Keywords:** Mollusca, Heterobranchia, kleptoplasty, lipidomics, phospholipids, glycolipids

## Abstract

**Simple Summary:**

Some species of sacoglossan sea slugs are able to steal chloroplasts from the algae they feed on and maintain them functional for several months, a process termed “kleptoplasty”. One of these photosynthetic slugs is *Elysia crispata*, found in coral reefs of the Gulf of Mexico. This sacoglossan inhabits different depths (0–25 m), being exposed to different food sources and contrasting light conditions. In this work, we characterized the pigment and fatty acid (FA) profiles, and quantified the total lipid, glycolipid and phospholipid contents of *E. crispata* from shallow (0–4 m) and deeper (8–12 m) waters, after a month of starvation to determine the longest and more stable retention of chloroplasts and its relation to habitat depth. Biochemical analyses allowed the identification of 12 photosynthetic pigments and 27 FAs. Heterogeneity in the composition of pigments confirmed the long-term retention of functional chloroplasts ingested from different algae. However, the differences found in pigment profile, total lipid content, and FA composition on individuals of *E. crispata* were not related to habitat depth. High amounts of glycolipids, exclusive chloroplast lipids, suggest a good condition of these photosynthetic organelles in animal cells. These results contribute baseline physiological data that may help explain evolutionary associations such as endosymbiosis.

**Abstract:**

Long-term retention of functional chloroplasts in animal cells occurs only in sacoglossan sea slugs. Analysis of molecules related to the maintenance of these organelles can provide valuable information on this trait (kleptoplasty). The goal of our research was to characterize the pigment and fatty acid (FA) composition of the sea slug *Elysia crispata* and their associated chloroplasts that are kept functional for a long time, and to quantify total lipid, glycolipid and phospholipid contents, identifying differences between habitats: shallow (0–4 m) and deeper (8–12 m) waters. Specimens were sampled and analyzed after a month of food deprivation, through HPLC, GC-MS and colorimetric methods, to ensure an assessment of long-term kleptoplasty in relation to depth. Pigment signatures indicate that individuals retain chloroplasts from different macroalgal sources. FA classes, phospholipid and glycolipid contents displayed dissimilarities between depths. However, heterogeneities in pigment and FA profiles, as well as total lipid, glycolipid and phospholipid amounts in *E. crispata* were not related to habitat depth. The high content of chloroplast origin molecules, such as Chl *a* and glycolipids after a month of starvation, confirms that *E. crispata* retains chloroplasts in good biochemical condition. This characterization fills a knowledge gap of an animal model commonly employed to study kleptoplasty.

## 1. Introduction

Superorder Sacoglossa includes sea slug species that are able to acquire chloroplasts from the algae they feed upon and keep them functional inside their digestive gland cells, a process termed kleptoplasty [1]. Until recently, sacoglossan sea slugs were the only known metazoans displaying such a mechanism [2]. Van Steenkiste et al. [2] reported the first known case of other metazoans (flatworms) capable of maintaining functional algal chloroplasts for one week. The retention period of “stolen” functional chloroplasts (kleptoplasts) within sacoglossan sea slugs may vary from a few hours to several weeks or months, depending on sea slug species, its life stage and the chloroplast algal source [3,4,5,6]. Moreover, abiotic factors such as irradiance levels and temperature can also play a key role over the time frame during which kleptoplasts are retained [7,8,9]. For instance, Vieira et al. [7] found that retention of photosynthetic activity of chloroplasts in starved *Elysia viridis* under high-light conditions decreased exponentially and lasted only 6 to 15 days, compared to low-light conditions in which retention times were longer (15 to 57 days) [7].

Photosynthetic sea slugs are generally stenophagous, feeding on a specific genus or species of algae. For example, *E. timida* only consumes algal species of genus *Acetabularia*, and *E. chlorotica* feeds exclusively on genus *Vaucheria* [10,11]. However, one of the exceptions to this stenophagous pattern is *E. crispata*, which can consume up to 17 different species of macroalgae according to DNA analyses [12]. The main genera of macroalgae this sacoglossan is known to feed upon are *Bryopsis*, *Penicillus* and *Halimeda* [12,13], although occasionally other genera are also mentioned [6,14,15]. *Elysia crispata* kleptoplast sources assessed through different techniques (e.g., electron microscopy, DNA barcoding) or observations are listed in Supplementary Table S1. Although *E. crispata* can feed on several species, only chloroplasts from the macroalgae *Bryopsis plumosa* and *Penicillus capitatus* have been reported to be kept functional for more than 10 weeks [16,17], while those originating from *Halimeda incrassata* were kept functional for up to 49 days [6]. In the case of *P. capitatus*, kleptoplasts were still present in slug tissues with different degradation levels after 120 days of starvation [18]. In another study, after one week of starvation of *E. crispata*, chloroplasts from four algal species were detected by DNA analysis: *P. capitatus*, *P. lamourouxii*, *H. incrassata* and *H. monile* [13]. Christa et al. [6] also analyzed the DNA of chloroplasts from *E. crispata* under 7, 28, 35 and 49 days of starvation and found different unidentified algae belonging to Bryopsidales, as well as the consistent presence of *H. incrassata* in the 8 sea slug specimens surveyed. The rest of macroalgal species reported through DNA barcoding (see Supplementary Table S1) were identified in sea slugs analyzed immediately after collection, without a period of starvation to clean their gut. As such, if chloroplasts from those food sources can also be retained functional for a long-term in this sacoglossan remains to be studied.

Many of the known photosynthetic pigments are light harvesting compounds present in chloroplasts, whose main function is to absorb the light energy necessary to power photosynthesis [19]. Pigment composition is highly variable depending on the type of algae and acclimation to the light regime [20]. Some pigments (i.e., xanthophylls) have been related to photoprotection, and it has been hypothesized that they may play an important role in kleptoplasts retention by sea slugs [21,22,23,24]. Chlorophyll *a* and *b* have been reported to be synthesized in sea slugs’ cells by some authors [25,26], while others have found no evidence supporting this synthesis [27]. Even though it is not clear if pigments are broken down or synthesized in the animal cells, they are still present after several days of starvation in different species of Sacoglossa. The presence of these molecules can be related to long-term retention inside sea slug cells because they may alleviate light-induced oxidative stress [20,22,23]. As such, the characterization of photosynthetic pigments present in kleptoplasts provides a baseline to better understand photoregulation in sea slugs inhabiting coastal marine environments [28,29].

Lipids and fatty acids (FAs) are a major source of metabolic energy, the main constituents of biological membranes, and play a relevant role in cell signaling [30]. In mollusks, these molecules are important for gametogenesis and can be used as an energy source during periods of food shortage [31]. The FA profile in mollusks and marine invertebrates depends on environmental and biological factors, such as diet, temperature or reproductive cycle [32]. Few works have related lipids to kleptoplasty in sacoglossan sea slugs. Pelletreau et al. [33] proposed that lipids synthesized by kleptoplasts inside *E. chlorotica* were crucial for the establishment and stabilization of kleptoplasty; moreover, Trench et al. [34] studied the incorporation of CO_2_ into glycolipids from the chloroplasts of *Codium fragile* to *E. viridis.* More recently, Rey et al. [35,36] analyzed the lipidome of two sea slug species, *E. viridis* and *Placida dendritica* feeding upon the same macroalgae, *C. tomentosum*, but with contrasting kleptoplasty performance. These studies revealed that the lipidome of the chloroplasts sequestered by *E. viridis*, which can perform long-term retention of functional chloroplasts, displays no major shifts and that the lipidome of this sea slug was unaffected by the absence of food for one week [35]. However, in *P. dendritica*, whose retention of chloroplasts is non functional, the lipidome varied significantly in this short period of time [36]. Therefore, information on composition and abundance of different lipid classes and FAs can help elucidate the importance and interaction of chloroplasts and animal cells.

*Elysia crispata*, the largest and most abundant species of Sacoglossa in the Caribbean and Gulf of Mexico, is one of the few species that can retain functional kleptoplasts for up to four months [1,6,13]. This sea slug can reach up to 15 cm in size [37], and inhabits mangrove areas and coral reefs from shallow water (<1 m) up to 25 m depth [38]. Different habitats at different depths provide diverse sources of macroalgal food, as well as contrasting environmental conditions, such as irradiance [39]. Distinct light conditions that change with depth can affect pigment composition and photosynthesis performance, and consequently, they may affect kleptoplasty as evidenced in experiments developed by Vieira et al. [7].

The goal of the present study was to characterize the photosynthetic pigment profile, total lipid, glycolipid-GL and phospholipid-PL contents and FA composition of *E. crispata* sampled at two habitat depths with different light conditions: shallow (0–4 m) and deeper (8–12 m) waters after a month of starvation to determine if the longest and more stable retention of chloroplasts is related to habitat depth. We hypothesized that the molecular composition of sea slugs varies with habitat depth.

## 2. Materials and Methods

### 2.1. Sample Collection

**Figure 1:**
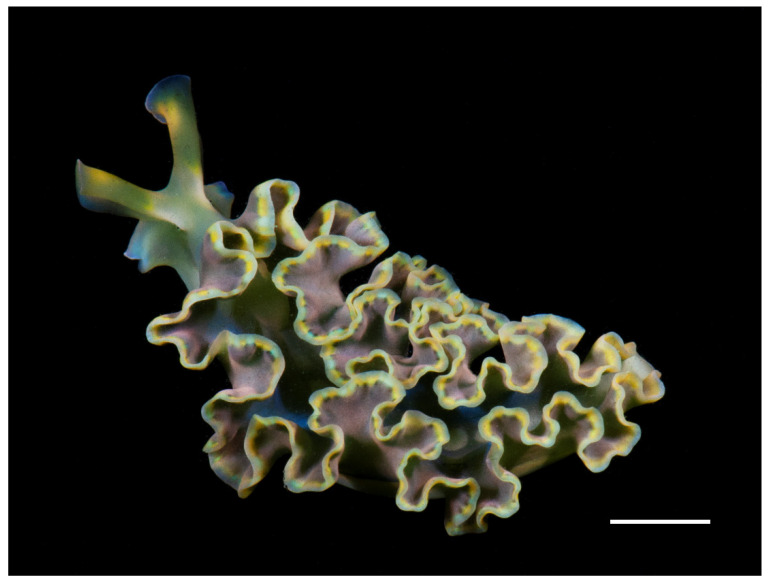
Specimen of *Elysia crispata* collected from the coral reef Verde (Sistema Arrecifal Veracruzano) in Veracruz, Southern Gulf of Mexico. Scale bar = 10 mm.

Specimens of *Elysia crispata* (Figure 1) from the same population were collected in three different sampling sites, less than 400 m apart from each other and with an approximate area of 300 m^2^, at the Verde coral reef (Sistema Arrecifal Veracruzano) in Veracruz, Southern Gulf of Mexico (19°12′8.80″ N, 96°4′17.20″ W) in a single day of September 2019. No macroalgae were spotted nearby, only turf algae were seen on top of hard substrate. Collecting was limited to the minimal sample size needed and was conducted under a permit issued by SAGARPA (PPF/DGOPA-082/19). Fourteen organisms measuring at least 40 mm in size (total length) were collected on hard substratum at 0–4 m depth (*n* = 7) and 8–12 m depth (*n* = 7). Their morphological characteristics matched the “crispata” ecotype described by Krug et al. [40]. Animals were transported to the laboratory, where they were maintained in a recirculating filtered seawater system (temperature: 26 °C, salinity: 35, light-dark photoperiod 12:12 h, with an irradiance of 40 µmol photons m^−2^ s^−1^). Animals were maintained for a month under starvation to ensure that sea slugs emptied their guts and digested short-term retained chloroplasts. Individuals were then flash frozen in liquid nitrogen and freeze-dried for biochemical analysis. The whole biomass of each sea slug was individually homogenized and used for extractions.

### 2.2. Photosynthetic Pigment Analysis

Photosynthetic pigments were extracted using methods detailed in Cruz et al. [29]. Briefly, samples were extracted in 95% cold buffered methanol with 2% ammonium acetate. Samples were ground with a plastic rod and sonicated for 30 s and vortexed. Samples were then transferred to −20 °C for 20 min in the dark. Extracts were filtered through 0.2 μm PTFE membrane filters and immediately injected into a Prominence-i LC 2030C High-performance liquid chromatography (HPLC) system (Shimadzu, Japan) with a photodiode array detector. Chromatographic separation was carried out using a Supelcosil C18 column (250 mm length; 4.6 mm diameter; 5 μm particles; Sigma-Aldrich, Burlington, MA, USA) for reverse phase chromatography and a 35 min elution program [41]. Photosynthetic pigments were identified from retention times and absorbance spectra, and concentrations calculated from the signals in the photodiode array detector. Pigment calibration was done using pure crystalline standards from DHI (Hørsolm, Denmark).

### 2.3. Lipid Analysis

Total lipids were extracted following a modification of the Bligh and Dyer method [42]. Briefly, animal tissues were homogenized, mixed with 800 µL of methanol and 400 µL of dichloromethane, vortexed and sonicated for 1 min, then, incubated on ice for 30 min on a rocking platform shaker and centrifuged at 2000 rpm for 10 min at room temperature. The organic phase was collected, and the biomass residue was re-extracted.

Ultrapure water was added (800 µL) and centrifuged at 2000 rpm for 10 min to recover the organic phase. An additional volume of 800 µL of dichloromethane was added to the aqueous phase, centrifuged at 2000 rpm for 10 min and the organic phase was recovered. Organic phases were dried under a nitrogen stream and preserved at –20 °C for further analysis. Total lipid extract weight was estimated by gravimetry.

Glycolipid (GL) quantification in total lipid extracts was performed using the orcinol colorimetric method [43]. Briefly, 200 µg of total lipid extract was transferred to a glass tube and 1 mL of orcinol solution (0.2% in 70% H_2_SO_4_) was added after dying dichloromethane under a nitrogen flow. Tubes were then incubated for 20 min at 80 °C. D-Glucose standards of 2–50 µg (standard solution of D-glucose 2.0 mg mL^−1^) were used to prepare the calibration curve. At room temperature, the absorbance of standards and samples was measured at 505 nm using a microplate UV-Vis spectrophotometer. The conversion factor 100/35 (ca. 2.8) was used to estimate the total GL content in total lipid extracts [44].

Phospholipid (PL) content from total lipid extract was quantified through the phosphorus assay, according to Bartlett and Lewis [45]. Lipid extracts were re-suspended in 300 µL of dichloromethane and 10 µL of each sample were transferred to a glass tube washed with 5% nitric acid. After drying under a nitrogen flow, 125 µL of perchloric acid (70%) was added and samples were incubated for 1 h at 180 °C in a heating block. A total of 825 µL of ultrapure water, 125 µL of NaMoO_4_∙H_2_O (2.5%), and 125 µL of ascorbic acid (10%) were added to each sample, with the mixture being homogenized in a vortex following each addition. Tubes were then incubated for 10 min at 100 °C in a water bath. Standards of 0.1–2.0 µg phosphate (standard solution of NaH_2_PO_4_∙2H_2_O, 100 µg of phosphorus mL^−1^) underwent the same treatment as samples, without the heating block step. At room temperature, absorbance of standards and samples was measured at 797 nm, using a microplate UV-Vis spectrophotometer. The conversion factor 775/31 (25) was used to estimate the total PL content in total lipid extracts.

FA profile of *E. crispata* was analyzed by gas chromatography-mass spectrometry (GC-MS). FA methyl esters (FAME) were prepared using 30 µg of total lipid extract, 1 mL of the internal standard 19:0 (0.5 µg mL^−1^ in *n*-hexane, CAS number 1731-94-8, Merck) and 200 µL of a methanolic solution of potassium hydroxide (2 M) [46]. After homogenization of this mixture, 2 mL of an aqueous solution of sodium chloride (10 mg mL^−1^) were added. Sample was centrifuged at 2000 rpm for 5 min to separate the phases. The organic phase containing the FAME was transferred to a microtube and dried under a nitrogen stream. FAME were then dissolved in 40 µL of *n*-hexane, and 2 µL of this solution were injected on an Agilent Technologies 6890 N Network chromatograph equipped with a DB-FFAP column with 30 m length, an internal diameter of 0.32 mm, and a film thickness of 0.25 µm (J&W Scientific, Folsom, CA, USA). The GC was connected to an Agilent 5973 Network Mass Selective Detector operating with an electron impact mode at 70 eV and scanning the mass range *m/z* 50–550 in 1 s cycle in a full scan mode acquisition. The initial oven temperature was 80 °C, staying at this temperature for 3 min and increasing linearly to 160 °C at 25 °C min^−1^, followed by linear increases to 210 °C at 2 °C min^−1^ and 250 °C at 30 °C min^−1^. Temperature was maintained at 250 °C for 10 min. The injector and detector temperatures were 220 °C and 250 °C, respectively. Helium was used as carrier gas at a flow rate of 1.4 mL min^−1^. FAME present in the sample were identified by comparing their retention time and mass spectra with a commercial FAME standard mixture (Supelco 37 Component FAME Mix, ref. 47885-U, Sigma-Aldrich) and confirmed by comparison with the spectral library from ‘The Lipid Web’ [47]. FAME were quantified by using calibration curves of FAME standards acquired under the same instrumental conditions [48].

### 2.4. Statistical Analysis

Total lipid, GL, PL and FA classes were analyzed through a Student’s *t*-test for independent samples, to assess if statistical differences existed between depths. Assumptions were verified through visual examinations (normal distribution) and Levene’s (homogeneity of variances) test [49,50]. Almost all of the distributions did not follow normality, thus, all of the data mentioned above (lipids, GL, PL and FA classes) were $log_{2}$ transformed, and then, the *t*-tests were performed [50].

To compare pigments and FA profiles of sea slugs from different depths, concentrations were first transformed to weight the contributions of all variables (√x was applied to pigments and log (*x* + 1) to FAs), then collinearity of variables was analyzed using a Draftsman Plot, and transformation of single variables was implemented whenever needed (log *x* + 0.1 for Anth and βε-Car, and log *x* for 16:2 *n*-6). Subsequently, all samples were normalized before analyses due to differences in the magnitude of values [51], and a principal component’s analysis (PCA) was performed for each data set to explore if any grouping occurred between organisms from the same depths. Subsequently, using these data, a resemblance matrix based on Euclidean distances among individual samples was made and used to perform a one-way analysis of similarities (ANOSIM), with depth as factor, for pigments and FAs separately, to test if statistically significant differences existed between depths. Data obtained in this study and used for analyses is available in https://doi.org/10.5281/zenodo.5398824 (Accessed on 25 October 2021). All procedures and graphs were performed using ggplot2 and stats packages in R v. 4.0.5 software [52], or PRIMER v.7 software [53]. Statistical significance was considered at *p* < 0.05.

## 3. Results

**Figure 2:**
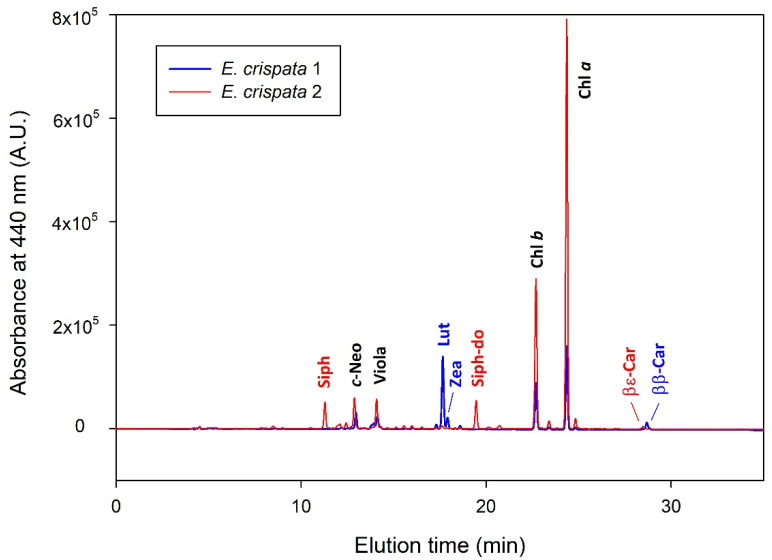
HPLC absorbance (440 nm) chromatograms of two specimens of *Elysia crispata* sampled at the same depth (0–4 m) and showing different photosynthetic pigment profiles. Common photosynthetic pigments are labeled in black, while specific photosynthetic pigments of samples 1 and 2 are shown in blue and red, respectively. See Table 1 for photosynthetic pigment abbreviations.

A total of 12 photosynthetic pigments were found in the samples of *E. crispata* (Table 1): 2 chlorophylls, 8 xanthophylls and 2 carotenes. While chlorophylls *a* and *b* (Chls *a* and *b*) were present in all individuals, the composition in carotenoids (xanthophylls and carotenes) differed between conspecifics. Some specimens displayed carotenoids typical of chloroplasts of siphonous green algae: siphonoxanthin (Siph), siphonoxanthin-dodecenoate (Siph-do) and β,ε-carotene (βε-Car); while others contained pigments from other algal types, such as lutein (Lut), zeaxanthin (Zea) and β,β-carotene (ββ-Car) (Figure 2); some specimens even displayed both sets of these carotenoids. To show examples of this pigment diversity, the profiles of two organisms from the same depth are represented in Figure 2.

**Figure 3:**
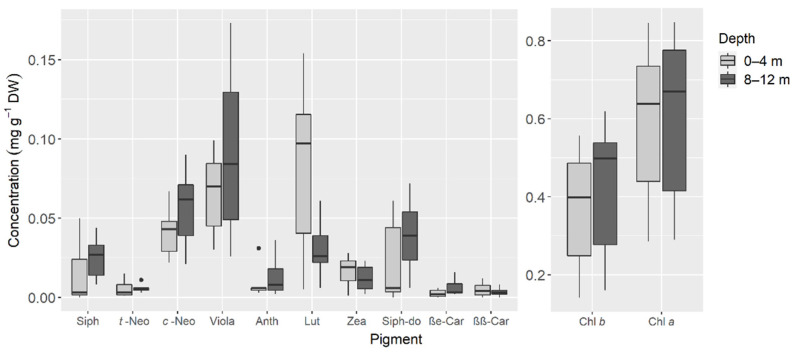
Concentration of photosynthetic pigments from samples of *Elysia crispata* collected at 0–4 m (*n* = 7) and 8–12 m (*n* = 7) depth, in the Southern Gulf of Mexico. The line represents the median, top and bottom of the box are the 25th and 75th percentiles. The whiskers represent the maximum and minimum values, and dots are outliers. See Table 1 for full names and respective abbreviations of pigments.

The concentration of photosynthetic pigments recorded in sea slugs originating from the two habitat depths is shown in Figure 3. Although some differences were observed, particularly for Lut (higher concentrations at the shallower habitat), differences in pigment profiles between individuals were not statistically related to habitat depth (ANOSIM: *p* = 0.305; R = 0.024).

**Figure 4:**
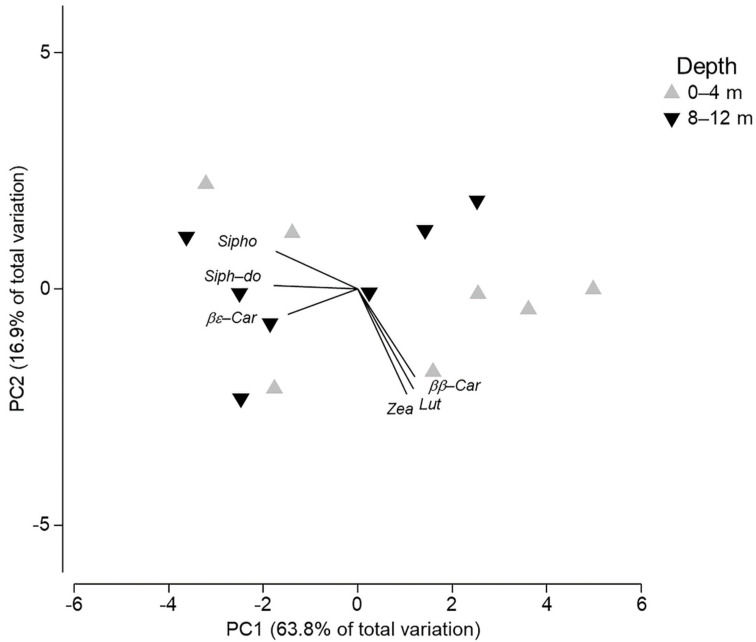
Principal components analysis (PCA) of photosynthetic pigment concentrations from samples of *Elysia crispata* collected at different depths in the Southern Gulf of Mexico.

Principal components analysis (PCA) of pigments did not exhibit grouping depending on depth (Figure 4). A total of 80.6% of the variation of pigment’s concentrations was explained by the first two components (PC1 63.8%, PC2 16.9%) (Figure 4). Most of the samples from 0 to 4 m habitats seemed to group in the right area of the *x* axis, but in general, samples were dispersed over both components. Vectors included in the graph show the different contribution of certain pigments in some of the individuals, as represented in Figure 2.

**Figure 5:**
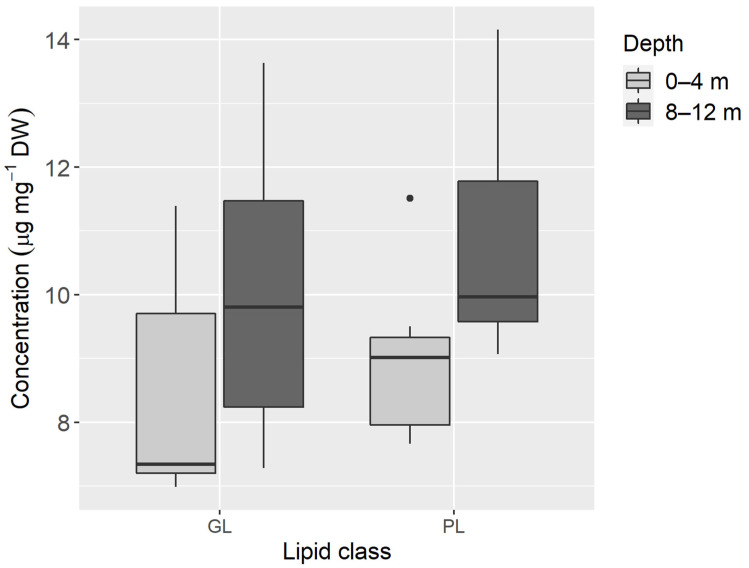
Glycolipid (GL) and phospholipid (PL) concentrations from samples of *Elysia crispata* collected at 0–4 m (*n* = 7) and 8–12 m (*n* = 7) depths, in the Southern Gulf of Mexico. The line represents the median, top and bottom of the box are the 25th and 75th percentiles. The whiskers represent the maximum and minimum values, and dots are outliers.

The amount of total lipid recorded averaged 79.0 ± 27.8 µg mg^−1^ DW (mean ± SD) in specimens collected from the shallow habitat (0–4 m) and 82.0 ± 17.1 µg mg^−1^ DW (mean ± SD) in those from the depth range of 8–12 m; no statistical differences were found between samples from both depths (t(8.437) = −0.496, *p* = 0.632). Likewise, GL and PL contents did not show statistical differences between depths (GL: t(11.87) = −1.346, *p* = 0.203; PL: t(11.36) = −2.134, *p* = 0.055), even though higher amounts (GL: 10.0 ± 2.3 µg mg^−1^ DW; PL: 10.8 ± 2.0 µg mg^−1^ DW) were found in the deeper habitat when compared to the shallow one (GL: 8.5 ± 1.9 µg mg^−1^ DW; PL: 8.9 ± 1.3 µg mg^−1^ DW) (Figure 5).

**Figure 6:**
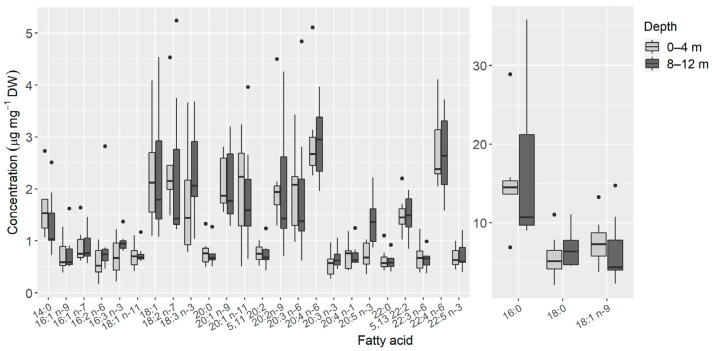
Fatty acid (FA) concentrations from samples of *Elysia crispata* collected at 0–4 m (*n* = 7) and 8–12 m (*n* = 7) depths, in the Southern Gulf of Mexico. The line represents the median, top and bottom of the box are the 25th and 75th percentiles. The whiskers represent the maximum and minimum values, and dots are outliers.

A total of 27 FAs were identified in the samples of *E. crispata* (Figure 6). Some variation was observed in the composition of FAs present in specimens collected at different depths, but also within those habitats. Therefore, no significant differences were found in the FA profiles between samples collected at 0–4 and 8–12 m depth (ANOSIM: *p* = 0.671; R = −0.046).

**Figure 7:**
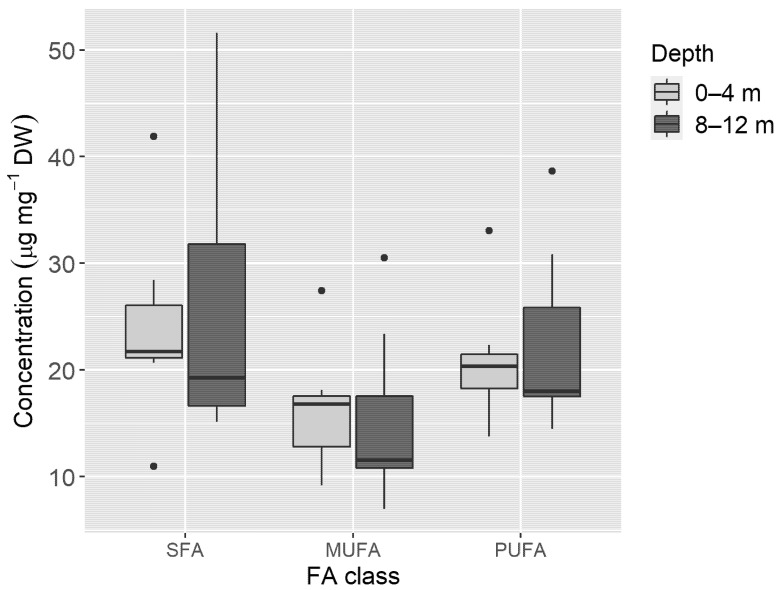
Concentrations of fatty acid (FA) classes from samples of *Elysia crispata* collected at 0–4 m (*n* = 7) and 8–12 m (*n* = 7) depths, in the Southern Gulf of Mexico. The line represents the median, top and bottom of the box are the 25th and 75th percentiles. The whiskers represent the maximum and minimum values, and dots are outliers. SFA—Saturated fatty acids; MUFA—Monounsaturated fatty acids; PUFA—Polyunsaturated fatty acids.

Saturated FAs (SFA) 16:0 and 18:0, as well as the monounsaturated FA (MUFA) 18:1 *n*-9 were the most abundant FAs found in samples from sea slugs sampled at both depths, with all other FAs displaying average values lower than 5 µg mg^−1^ DW (Figure 6). Of the FAs found in these sea slugs, 5 were SFA, 7 were MUFA and 15 were polyunsaturated FAs (PUFA). SFA represented almost 40% of the FA content, while PUFA (35%) and MUFA (25%) levels were lower. None of the FA classes showed statistically significant differences with depth (SFA: t(11.769) = −0.201, *p* = 0.843; MUFA: t(10.577) = 0.615, *p* = 0.551; PUFA: t(11.18) = −0.287, *p* = 0.779), despite higher mean values of SFA (26.1 ± 13.4 µg mg^−1^ DW) and PUFA (22.5 ± 8.8 µg mg^−1^ DW) being recorded in specimens collected at the deeper habitat (Figure 7). MUFA was the only FA class with slightly higher mean values in the shallower habitat at 0–4 m (16.3 ± 5.8 µg mg^−1^ DW), compared to the 8–12 m depth (15.1 + 8.5 µg mg^−1^ DW).

**Figure 8:**
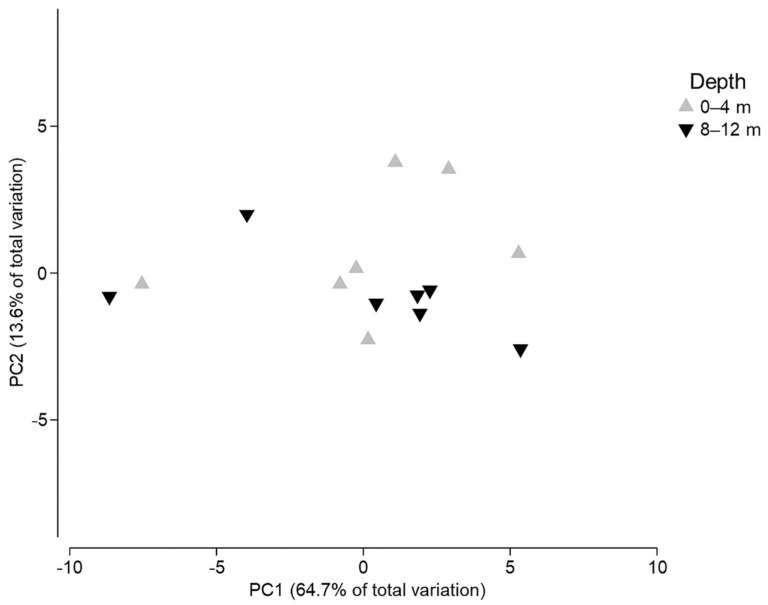
Principal components analysis (PCA) of fatty acid (FA) concentrations from samples of *Elysia crispata* collected at different depths in the Southern Gulf of Mexico.

Finally, the PCA showed that a total of 78.3% of the variation of FA’s concentrations was explained by the first two components (PC1 64.7%, PC2 13.6%) (Figure 8). However, almost all FAs contributed to the distribution of samples, as no grouping was observed. Figure 8 is a good representation of the ANOSIM analysis results mentioned previously, where differences were not statistically related to habitat depth.

## 4. Discussion

In the present study, we provided a characterization of pigments, FAs and lipid classes (PL and GL) from *E. crispata* sampled at different depths, which helps to fill a knowledge gap of one of the animal models most commonly employed to study kleptoplasty. To our knowledge, this is the first effort assessing differences in depth for a population of a sacoglossan sea slug, and the first work in the most western distribution recorded for this species. Concentrations of pigments were heterogeneous over different specimens of this sea slug and were not related to habitat depth.

A chloroplast photosynthetic pigment profile was previously described for specimens of *E. crispata* collected in Florida, where authors found matching profiles between *Caulerpa sertularioides* and sea slugs, with the exception of the presence of two subproducts in the sea slug: phaeophorbide *a* and phaeophytin *a* [54]. These pigments are chlorophyll breakdown intermediates [55] and were not found in our samples. Chlorophyll synthesis in sea slug tissues has been reported to occur in *E. chlorotica* [25], and *E. crispata* [26]; however, Trench and Smith [27] stated that this mechanism was unlikely to occur in *E. crispata* and *E. viridis*, as they only detected small traces of Chl *a* and *b* in both species [27]. These dissimilarities could be due to the macroalgal origin of kleptoplast or differences in technique employed to perform this assessment. Similarly, Trench et al. [34] did not find evidence of chlorophyll synthesis in *E. viridis* after 24 h of starvation [34]. Studies in other long-term retention species have reported Chl *a* values of 0.3 mg g^−1^ DW after 2 weeks of starvation in *E. viridis* [29] and <0.4 mg g^−1^ DW after 28 days of starvation in *E. timida* [56]; here, we found mean values of almost 0.6 mg g^−1^ DW after 1 month of starvation in *E. crispata*. Thus, this higher amount of Chl *a*, together with the absence of Chl *a* breakdown products, support the apparent good condition of kleptoplasts within the cells of this sea slug species.

Photosynthetic pigment analysis of three species of *Elysia* has shown that their profile is almost identical to their food source, *E. viridis*-*C. tomentosum*, *E. timida*-*Acetabularia acetabulum* and *E. chlorotica*-*Vaucheria litorea*, except for an unidentified carotenoid in the first two *Elysia* species [11,21,22,23,24,29,48,57]. The photosynthetic pigment composition recorded in *E. crispata* in the present study not only reflects the different macroalgal origins of acquired kleptoplasts but, most importantly, it also shows that after a month of food deprivation, these sea slugs conserved the heterogeneity of their photosynthetic pigment profiles and the relatively high values of Chls *a* and *b*. Curtis et al. [13,17] working with *E. crispata* provided evidence of a simultaneous chloroplast sequestration of at least two macroalgal species (*P. capitatus* and *B. plumosa*) for several weeks [13,17], while Christa et al. [6] found that kleptoplasts originating from *H. incrassata* could be retained for up to 49 days. These last authors assessed *E. crispata*‘s food sources through DNA barcoding and signaled the presence of several unidentified algae from order Bryopsidales retained for 7, 28, 35 and 49 days, as well as *Acicularia schenkii* (found in one starved slug for 7 days) and *A. acetabulum* (found in a non-starved individual) [6]. Only these last two species belong to the group of algae with a pigment profile that includes lutein, antheraxanthin, zeaxanthin, or ββ-Carotene [11], a profile matching those recorded in some specimens surveyed in the present study. Thus, the present work confirms that the long-term retention of kleptoplasts from this type of algae does occur in *E. crispata*.

Unlike photosynthetic pigments, whose origin in Sacoglossa sea slugs is exclusive from sequestered chloroplasts, the FA profiles recorded are biosynthesized by animal metabolism and/or have origin in their macroalgal diet (including the digested cytoplasmic content and the kleptoplasts retained). However, GL are exclusive lipids of chloroplast membranes [58]; thus, finding high concentration of GL in *E. crispata* after 1 month of starvation confirms the preservation of chloroplast membranes integrity inside sea slug cells. *Elysia crispata* shows a similar concentration of PL and GL (Figure 5); nevertheless, in *E. viridis* and *P. dendritrica* the concentration of PL is higher than GL under starved or fed conditions [36]. This could be due to the larger size of *E. crispata*, when compared to *E. viridis* and *P. dendritica*, as well as the foliose shape of its parapodia, hence allowing *E. crispata* to harbour a higher number of kleptoplasts in proportion to its body size. Analyses of lipids and FAs, as performed before for *E. chlorotica, E. viridis* and *E. timida*, could be important for estimating kleptoplast energetic contribution for the animal and their specific support in reproductive fitness [33,36,48,59]. To understand if something similar occurs in *E. crispata*, further studies using isotope labeling are still required [36].

We acknowledge that sample size may have contributed for no statistically significant differences having been recorded. Unfortunately, we were not able to collect more organisms due to logistic constraints in the field and the laboratory. Therefore, we suggest that a higher number of specimens should be sampled in future studies addressing *E. crispata*, as this species presents high intraspecific variability. The amount of PL recorded between specimens collected at different depths did not show significant statistical differences according to the *t*-test (*p* = 0.055); such a marginal value should not be considered conclusive. PL are the main lipids in animal cell membranes and thus, are quite adaptive to specific environmental conditions. In the study area, several physical and chemical parameters can change in a few meters of the water column depending on the season [60,61]. Differences in PL could be present between habitats, but not detected because they occur in a gradient or in a distinct depth range. Then, differences in PL should not be completely discarded.

No other studies have addressed a characterization of FAs in *Elysia crispata*. FA composition is influenced by external (i.e., environmental) conditions and diet. Most FAs are esterified in different lipid classes, such as polar lipids and triglycerides, which play diverse roles (e.g., structural, signaling, energy reserves). Previous lipidomics studies in marine sacoglossan sea slugs and macroalgae have identified the molecular composition of different polar lipids, such as PL (e.g., phosphatidylcholine [PC], phosphatidylethanolamine [PE]) and GL (e.g., sulfoquinovosyl diacylglycerol [SQDG], monogalactosyl diacylglycerol [MGDG], digalactosyl diacylglycerol [DGDG]), providing information at molecular level on their structural and fatty acyl chains composition [35,36]. The high concentration of 16:0 in this group of sea slugs must be related with its structural role in membrane lipids, since this FA is usually esterified in GL and PL. For example, the most abundant SQDG molecular species recorded in *E. viridis* were SGDG (16:0/16:0), SQDG(18:3/16:0) and SQMG (16:0), characteristic lipids of chloroplast membranes [35]. Both 16:0 and 18:0 occur as FAs esterified in several PL molecular species of *E. viridis* such as PC 36:4, PC 38:4, PC 38:5, PE 38:2, PE 38:3, PE 38:4 and PE 38:5 [36]. In addition, the high content of PUFA is probably derived from dietary items. *Elysia crispata* had high amount of GL, and could have a similar composition with *E. viridis*, who presented MGDG and DGDG, with a high proportion of 16:3 and 18:3 [35].

FAs such as 20:5 *n*-3 (EPA), 22:6 *n*-3 (DHA) and 20:4 *n*-6 (ARA) are essential for marine organisms. Although recent studies have found that several marine groups possess desaturase genes that allow them to produce PUFA de novo [62], most marine invertebrates and fish cannot biosynthesize enough *n*-3 PUFA to cover their physiological demand. As such, most of these marine organisms rely on their diets as the main source to acquire these biomolecules [31,63]. Many marine mollusks have EPA and DHA accounting for half of their total FAs [32]. However, in our results, only 20:5 *n*-3 was found, and in low amounts (Figure 6). In 0–4 m samples, we identified the characteristic EPA fragmentation in the MS spectrum at its retention time (21.6 min in standard mix); in 8–12 m, the MS spectrum at the same retention time showed some fragment ions characteristic of EPA, however, the most abundant fragment ions correspond to an unknown compound. Hence, the abundance of this FA must be interpreted with caution in sea slugs sampled at the 8–12 m depth, because this FA might be overestimated due to the presence of another unknown compound with a similar retention time.

The lack of DHA in *E. crispata* samples may be related to the absence of this FA in its food sources. Although this is a common FA in marine organisms, it is absent in macroalgae recognized as common food sources of sacoglossan sea slugs, such as *C. tomentosum* [64], and *A. acetabulum* [59]. Moreover, the most abundant PUFA in *E. crispata* was 20:4 *n*-6. In other sea slugs without kleptoplasty, such as nudibranchs and cephalaspideans, high amounts of ARA have also been recorded [65,66]. Therefore, these high amounts of ARA may be phylogenetically driven and part of the natural FA profile of sea slugs [66,67].

The amount of FA classes depends on gastropod species [31]. Some gastropod herbivores have higher amounts of PUFA, for instance, in *Littorina littorea* these FAs account for 52% of total lipids [32], and in *E. timida* represents 57% [59]. In this study, PUFA in *E. crispata* represents around 35% of the total pool of FAs, while in chromodorid nudibranchs, which feed on sponges, it accounts for 15–31% [65]. Low proportions of SFA (8-16%) can be found in chromodorid nudibranchs. In contrast, SFA in *Armina* nudibranchs, which feed on cnidarians [66], *Patella peroni,* a gastropod herbivore [32], and *E. crispata* correspond to around 40% of total lipids. As diet influences FA composition, the FA profiles mentioned above, and its proportions are most likely explained by dietary items. Nudibranchs have FAs similar to their sponge preys [65], and *E. viridis* has a glycolipid profile very similar to its food source, *C. tomentosum*, due to the preservation of chloroplasts in their cells [35,36].

To date, 25 species of macroalgae have been reported as potential food sources for *E. crispata* (Supplementary Table S1). Some authors recommend considering only the 17 species confirmed by DNA molecular analysis [12]. Apparently, its preferred dietary items are those within genera *Bryopsis*, *Penicillus,* and *Halimeda*, while consumption of genus *Caulerpa* has triggered controversy [12,40,68], even when the photosynthetic pigment profiles of this macroalgae match those of this sea slug [54]. For a different project, our own preliminary observations in the laboratory suggest *E. crispata* from the same population of the present study does not consume *Udotea*, *Ulva*, *Rhipocephalus* and *Caulerpa*. The specimens of *E. crispata* sampled in the present study retained kleptoplasts from Siphonales (the group of algae recognized as its favorite diet), as evidenced by the presence of siphonoxanthin and siphonoxanthin-dodecenoate; nonetheless, *E. crispata* also showed kleptoplasts from another group of macroalgae displaying lutein as their main xanthophyll (e.g., *Acetabularia*). This last pigment was relatively higher in sea slugs from shallower waters. As higher light levels occur in shallower environments, this pigment may be involved in photoprotection [69].

Light availability can drastically change in the coral reef where *E. crispata* was studied. For example, light intensity in a nearby reef (Anegada de Adentro) at 10 m depth only represents about 15% of the light levels recorded at 1 m depth [70]. Moreover, in another close by reef (Sacrificios) during the same season (August–September) as that during which the collection of specimens for the present study was performed, light intensity at 19 m depth varied from 1000 to 9000 Lux [71], an equivalent of ∼18.5–166.5 μmol photons m^−2^ s^−1^ [72]. This suggests that the amount of light recorded in shallow habitats in these coral reefs can be very high; however, our results did not support differences in the acclimation state of kleptoplasts of sea slugs sampled in such distinct habitats. As longer distances between sampling depths (>4 m) might have helped to detect some differences, this issue should be considered in future works.

Long-term retention of chloroplasts in sacoglossans depends on sea slug species and the macroalgae they feed upon [3,4,6]. The longest retention time of kleptoplasts in *E. crispata* has been reported as 120 days [18], and here, we confirm a good physiological and biochemical condition of chloroplasts after 30 days. Our results support that *E. crispata* digests its kleptoplasts slowly and that, during long starvation periods (one month) chloroplast diversity remains [17,18]; in general, the heterogeneity observed in pigments, lipids and FAs was not related to habitat depth. More basic research on this sacoglossan is required to understand kleptoplasty and explain evolutionary associations, such as endosymbiosis.

## 5. Conclusions

The heterogeneity recorded in the profile of photosynthetic pigments and FA composition of *E. crispata* was not related to the habitat depth at the coral reef where they were sampled in the Southern Gulf of Mexico. The total lipid, PL and GL contents found in this work were similar for specimens collected at shallow (0–4 m) and deeper (8–12 m) habitats. The conserved heterogeneity of their photosynthetic pigment profiles, as well as the high content of molecules exclusive of chloroplasts recorded on *E. crispata*, such as Chl *a* and GL after a month of food deprivation confirms that these sea slugs retain chloroplasts in good condition for long periods of time after stealing them from macroalgae.

## Figures and Tables

**Table 1:** Photosynthetic pigments found in samples of *Elysia crispata* collected in Southern Gulf of Mexico (Sistema Arrecifal Veracruzano), with retention times and absorption maxima (λ max).

Pigment	Retention Time (min)	λ max (nm)
Siphonoxanthin (Siph)	11.38	448
all-*trans*-neoxanthin (*t*-Neo)	12.46	440, 471
*cis*-neoxanthin (*c*-Neo)	12.98	414, 438, 467
Violaxanthin (Viola)	14.21	418, 442, 471
Antheraxanthin (Anth)	16.18	448, 476
Lutein (Lut)	17.86	448, 476
Zeaxanthin (Zea)	18.11	453, 480
Siphonoxanthin-dodecenoate (Siph-do)	19.76	454
Chlorophyll *b* (Chl *b*)	23.09	458, 646
Chlorophyll *a* (Chl *a*)	24.71	431, 663
β,ε-Carotene (βε-Car)	29.01	449, 477
β,β-Carotene (ββ-Car)	29.22	454, 478

## Data Availability

Data supporting reported results can be found at https://doi.org/10.5281/zenodo.5398824.

## Supplementary Materials

The following are available online at https://www.mdpi.com/article/10.3390/ani11113157/s1, Table S1: Algae consumed by Elysia crispata and its presence in the study area, according to literature [1,6,12,13,14,15,16,40,54,68,73,74,75,76,77,78,79,80,81].
