# Supplementary material for: Pigment and Fatty Acid Heterogeneity in the Sea Slug Elysia crispata Is Not Shaped by Habitat Depth
Source: Animals (Basel). 2021 Nov 5;11(11):3157. doi: 10.3390/ani11113157 (PMC8614334; doi:10.3390/ani11113157)
Supplement: Supplementary file 1 [file animals-11-03157-s001.zip › animals-1413664-supplementary.pdf]

Supplementary material to the publication: Pigment and fatty acid heterogeneity in the sea slug *Elysia crispata* is not shaped by habitat depth.

Xochitl G. Vital [1,2], Felisa Rey [3,4*], Paulo Cartaxana[5], Sónia Cruz[5], M. Rosário Domingues[3,4], Ricardo Calado[5] and Nuno Simões [2,6,7*]

Table S1. Algae consumed by *Elysia crispata* and its presence in the study area, according to literature. Presence of algae in collecting site of this study (Sistema Arrecifal Veracruzano) according to Galicia-García et al. [73].

Algae	Origin of slugs (collected or cultured)	Assessment	References	Present in collecting site (Verde reef)
Acetabularia crenulata	col. Florida	DNA	[6]	YES
Acicularia schenckii	col. Florida	DNA	[6]	YES
Batophora oerstedi	col. Florida	observations	[14]	NO
***Batophora* sp.**	not mentioned	observations	[6[a],15[b]]	NO
***Bryopsidella* sp.**	col. Florida	DNA	[68] in [12]	NO
Bryopsis pennata	col. Florida, Virgin Islands, Panama	DNA	[12,68,74]	YES[c]
Bryopsis pennatula	col. Florida	DNA	[68]	NO
Bryopsis plumosa	col. Florida; cultured	observations; microscopy and DNA	[14,15,74,75]	YES[c]
***Bryopsis* spp.**	col. Florida, Curacao, Virgin Islands, Panama	DNA; observations	[6,12,40,68]	Only the species mentioned above[c]
Caulerpa ambigua	col. Panama	DNA	[12]	NO
Caulerpa racemosa	col. Florida & Jamaica	observations	[76]	YES
Caulerpa sertularioides	col. Florida & Jamaica	pigment analysis (HPLC); observations	[54,76,77]	YES
***Caulerpa* spp.**	col. Florida	DNA; observations	[6 [a],14[d]]	YES, additionally *C. cupressoides*
Caulerpa verticillata	col. Florida & Jamaica	observations	[1,14,15,76,78]	NO

***Chaetomorpha* sp.**	col. Florida & Jamaica	observations	[76]	YES, only *C. nodosa*
***Chlorodesmis* sp.**	col. Curacao, Florida	DNA	[6[a],12]	NO
***Codium* sp.**	not mentioned	not mentioned	[79[e]]	YES, only *C. taylorii*
***Cymopolia* sp.**	not mentioned	not mentioned	[40[f]]	YES
***Derbesia* sp.**	col. Florida, Panama	DNA	[12,68,74]	NO
Derbesia tenuissima	cultured	electronic microscopy and observations	[74]	NO
Halimeda discoidea	col. Florida & Jamaica	observations	[76]	YES
Halimeda incrassata	col. Florida & Virgin Islands	DNA and microscopy	[6,12,13,68,75]	NO
Halimeda monile	col. Florida	DNA	[6,13,68]	NO
Halimeda opuntia	col. Virgin Islands	DNA	[75]	YES
Halimeda simulans	col. Panama	DNA	[12]	NO
Halimeda tuna	col. Florida	DNA	[12]	YES
***Halimeda* spp.**	col. Florida, Panama	observations; DNA	[6,12,14,15,68]	YES, additionally *H. scabra*
Parvocaulis exiguus	col. Curacao, Panama	DNA	[12]	NO
Penicillus capitatus	cultured; col. Florida & Virgin Islands	electronic microscopy; DNA; observations	[6,13,16,68,74,75]	NO
Penicillus dumetosus	not mentioned	DNA	[6[a]]	NO
Penicillus lamourouxii	col. Florida	DNA	[6[a],13, 68]	NO
Penicillus pyriformus	col. Florida	DNA	[68]	NO
***Penicillus* sp.**	col. Florida	DNA; observations	[6[a],14,15]	NO
Polyphysa peniculus	col. Florida	DNA	[12[g]]	NO
***Pseudochlorodesmis* sp.**	col. Florida	DNA	[6[a], 68]	NO
**Pseudocodiaceae sp.**	col. Florida	DNA	[6]	NO

**Rhipiliaceae sp. 1**	col. Florida	DNA	[6]	NO
Rhipocephalus phoenix	col. Florida	observations	[14[d]]	YES
***Siphonogramen* spp.**	col. Curacao, Virgin Islands, Florida, Panama	DNA	[12]	NO
**Udoteaceae**	col. Virgin Islands, Curacao & Florida, Panama; Florida	DNA	[12,74,75]	YES, only *R. phoenix*
**Ulvophyceae sp. 1**	col. Florida	DNA	[6]	YES, *Ulva compressa, U. flexuosa, U. intestinalis, U. lactuca, U. paradoxa*
Vaucheria litorea	not mentioned	not mentioned	[80[h]]	NO

[a]Included in the list provided by its Supp. Data 2 [6].
[b]Reference [15] compiled information from literature up until that year; however, included her personal observations.
[c]According to [81], *Bryopsis pennata* is the only valid species in the Atlantic coast of Mexico.
[d]Authors mention that results regarding the feeding of this species of algae were inconclusive.
[e]This publication does not provide detailed information, only states "Certain marine slugs such as *Tridachia crispata* and *Elysia viridis* feed on siphonaceous seaweeds such as *Codium*".
[f]Could not be tracked in the publications cited by those authors and [78]; thus, authors probably observed slugs feeding on this algae.
[g]Reference [12] cites this species from reference [68], but we were not able to find *Polyphysa peniculus* in the original reference.
[h]This publication does not provide detailed information, only states "*Vaucheria litorea*, an alga eaten by *E. chlorotica* and, according to our feeding experiments, by juveniles of *E. crispata*".
